# Functional Disability and Psychological Impact in Headache Patients: A Comparative Study Using Conventional Statistics and Machine Learning Analysis

**DOI:** 10.3390/medicina61020188

**Published:** 2025-01-22

**Authors:** Jong-Ho Kim, Hye-Sook Kim, Jong-Hee Sohn, Sung-Mi Hwang, Jae-Jun Lee, Young-Suk Kwon

**Affiliations:** 1 Department of Anesthesiology and Pain Medicine, Chuncheon Sacred Heart Hospital, Hallym University College of Medicine, Chuncheon 24253, Republic of Korea; poik99@hallym.or.kr (J.-H.K.); h70sm@hallym.or.kr (S.-M.H.); 2 Institute of New Frontier Research, Hallym University College of Medicine, Chuncheon 24253, Republic of Korea; hskim87@hallym.ac.kr (H.-S.K.); deepfoci@hallym.or.kr (J.-H.S.); 3 Big Data Center, Chuncheon Sacred Heart Hospital, Hallym University College of Medicine, Chuncheon 24253, Republic of Korea; 4 Department of Neurology, Chuncheon Sacred Heart Hospital, Hallym University College of Medicine, Chuncheon 24253, Republic of Korea

**Keywords:** headache, pain intensity, depression, anxiety, functional disability, machine learning, linear models

## Abstract

*Background and Objectives*: Recent research has focused on exploring the relationships between various factors associated with headaches and understanding their impact on individuals’ psychological states. Utilizing statistical methods and machine learning models, these studies aim to analyze and predict these relationships to develop effective approaches for headache management and prevention. *Materials and Methods*: Analyzing data from 398 patients (train set = 318 and test set = 80), we investigated the influence of various features on outcomes such as depression, anxiety, and headache intensity using machine learning and linear regression. The study employed a mixed-methods approach, combining medical records, interviews, and surveys to gather comprehensive data on participants’ experiences with headaches and their associated psychological effects. *Results*: Machine learning models, including Random Forest (utilized for Headache Impact Test-6, Patient Health Questionnaire-9, and Generalized Anxiety Disorder-7) and Support Vector Regression (applied to Migraine Disability Assessment), revealed key features contributing to each outcome through Shapley values, while linear regression provided additional insights. Frequent analgesic medication emerged as a significant predictor of poorer life quality (Headache Impact Test-6, root mean squared error = 7.656) and increased depression (Patient Health Questionnaire-9, root mean squared error = 5.07) and anxiety (Generalized Anxiety Disorder-7, root mean squared error = 4.899) in the Random Forest model. However, interpreting the importance of features in complex models like supportive vector regression poses challenges, and determining causality between factors such as medication usage and pain severity was not feasible. *Conclusions*: Our study underscores the importance of considering individual characteristics in optimizing treatment strategies for headache patients.

## 1. Introduction

Headaches are a prevalent symptom worldwide, causing significant discomfort for many individuals [1]. This discomfort is often associated with psychological issues and can lead to mental health problems such as depression and anxiety disorders [2,3,4]. Additionally, the functional impairment resulting from headaches can diminish the quality of life for individuals and incur social and economic costs [5,6,7,8].

Secondary headaches are usually caused by specific conditions or diseases, while primary headaches often have unknown underlying causes. However, it is believed that dysfunction of the nerves, blood vessels, and chemical imbalances are involved [9]. Migraines occur periodically and are often accompanied by nausea, vomiting, and sensitivity to light and sound [10]. Tension-type headaches are associated with stress and muscle tension [11]. Physiological changes, such as hormonal shifts or sleep pattern disturbances; environmental factors, such as noise, light, and smells; dietary factors, such as caffeine and certain foods; and psychological factors, such as stress, can act as triggers [12,13,14]. If treatment is insufficient or poorly managed, these triggers can also become persistent factors [15,16,17].

Among patients complaining of headache, the prevalence of depression and anxiety and depression has been reported to be 19.7% and 13.7%, respectively [18]. In particular, migraine is associated with an increased risk of anxiety and depression, significant disability, and a reduced quality of life. Furthermore, an increase in monthly headache is linked to greater disability and lower quality of life [19]. The relationship between anxiety disorders and migraines is bidirectional, as recurrent migraine can lead to anxiety, and anxiety can, in turn, exacerbate migraines [20].

Previous research has illuminated the relationship between headaches and mental health issues and has utilized various statistical techniques to measure the extent of functional impairment due to headaches [3,21,22]. However, traditional statistical methods may have limitations in fully understanding the complexity of the data and the intricate relationships between factors. Recent advancements in technology have demonstrated remarkable success in discovering patterns in data and understanding complex interactions through artificial intelligence and machine learning [23,24,25,26]. The capacity of machine learning methods to process large and complex datasets makes them well suited for application in precision medicine. Machine learning techniques, as previously utilized in the medical field, can be employed to investigate the relationships between headache-related factors, psychological effects, and functional impairments. Additionally, by utilizing various types of clinical data, they can predict patients’ physiological outcomes and inform treatment strategies. From this perspective, we propose advancing methods to further leverage machine learning to optimize patient care [27,28]. Also, leveraging these novel technologies to evaluate the mental health status of headache patients and assess the functional impairment caused by headaches is crucial. In this study, we will utilize both conventional statistical techniques and machine learning models to evaluate factors associated with headaches and compare key factors such as depression, anxiety, headache intensity, and functional impairment due to headaches. It is anticipated that this research will contribute to providing personalized treatment and management strategies for headache patients.

## 2. Materials and Methods

### 2.1. Study Design

This study is part of the ongoing “Development of quantification and management system on pain-using artificial intelligence-based multi-modal registration technology” research project, and the main goal of this study was to explore the correlation between headache-related factors, psychological effects, and dysfunction using both traditional statistical methods and machine learning models. Additionally, we aimed to compare the key factors identified by each model. Patients included in this study were sourced from the “Development of quantification and management system on pain using artificial intelligence-based multi-modal registration technology” registered in the Korean Clinical Trial Database (http://cris.nih.go.kr/cris/index/index.do; KCT0008684; accessed on 21 February 2024). The insights gained from our investigation will be instrumental in advancing the ongoing “Development of quantification and management system on pain using artificial intelligence-based multi-modal registration technology”. The inclusion criteria encompassed adult men and women aged 19 to 80 seeking treatment for headaches at Chuncheon Sacred Heart Hospital between 1 April 2023 and 13 February 2024, including both outpatients and inpatients, as well as those attending the emergency room. The causes of headaches included various conditions such as migraines (with or without aura), status migrainosus, complicated migraines, and other or unspecified types of migraines. Additionally, acute, chronic, episodic, and unspecified tension-type headaches (TTH) were considered, along with other specified cerebrovascular diseases, other headache syndromes, dizziness, giddiness, benign intracranial hypertension, vascular headaches, cluster headache syndrome, meningitis, viral meningitis, post-concussion syndrome, herpes zoster without complications, and unspecified headaches. Exclusion criteria ruled out individuals who refused to participate, those unable to express their intentions due to mental or physical impairments, those incapable of completing surveys or answering questions, and individuals younger than 19 or older than 80 years. The detailed inclusion and exclusion criteria for this study can be found in a previous study that used data from the original project [29].

### 2.2. Ethical Approval and Informed Consent

This study was approved by the Chuncheon Sacred Heart Hospital Institutional Review Board (IRB No. 2022-08-002-001). All research activities strictly adhered to the ethical principles outlined in the Helsinki Declaration. Written informed consent was obtained from all participants.

**Figure A1:**
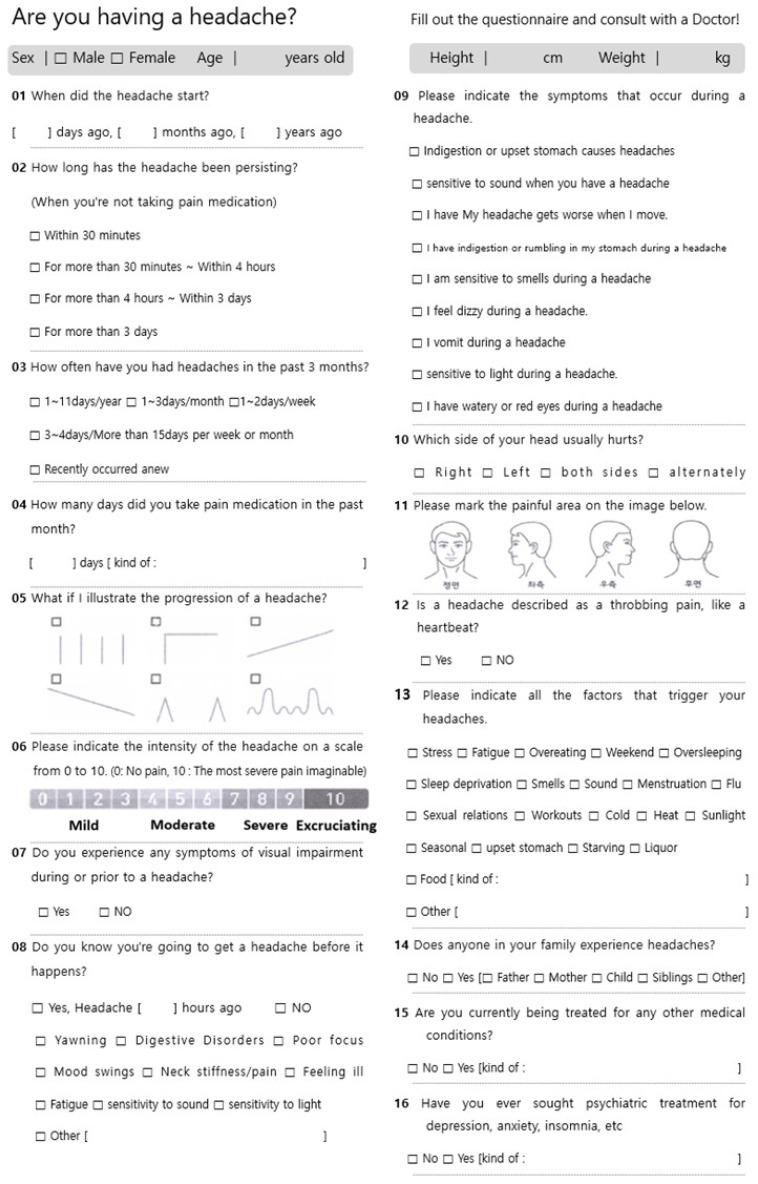
Headache survey.

**Figure A2:**
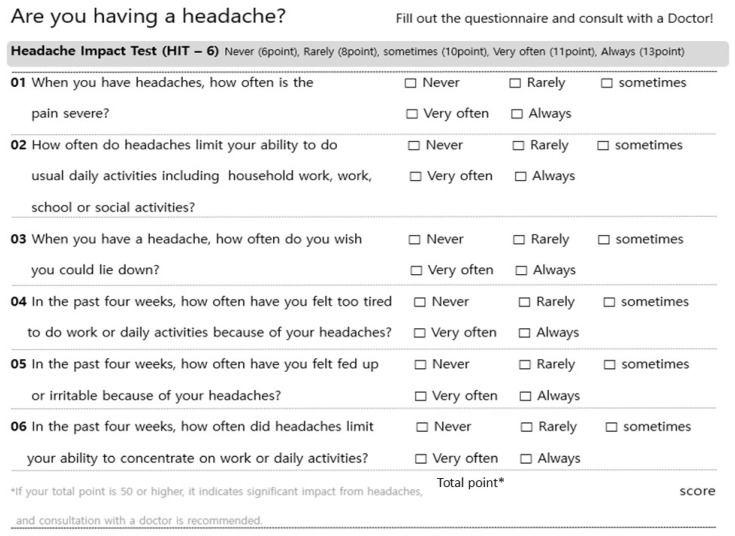
Headache Impact Test-6.

**Figure A3:**
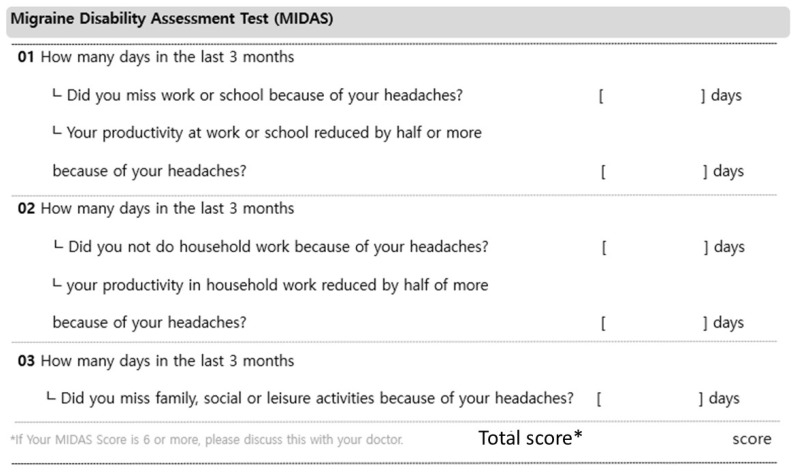
Migraine Disability Assessment.

**Figure A4:**
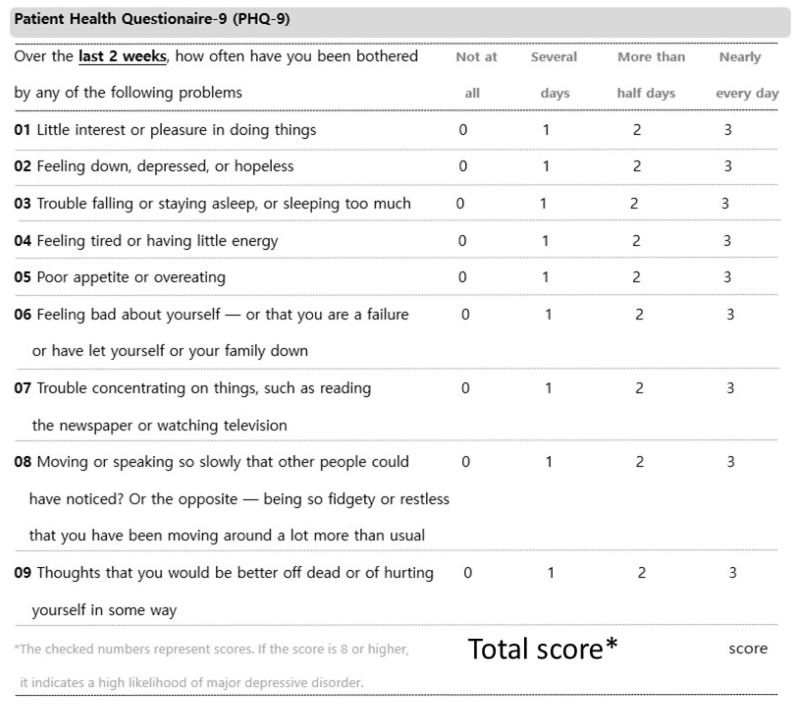
Patient Health Questionnaire-9.

**Figure A5:**
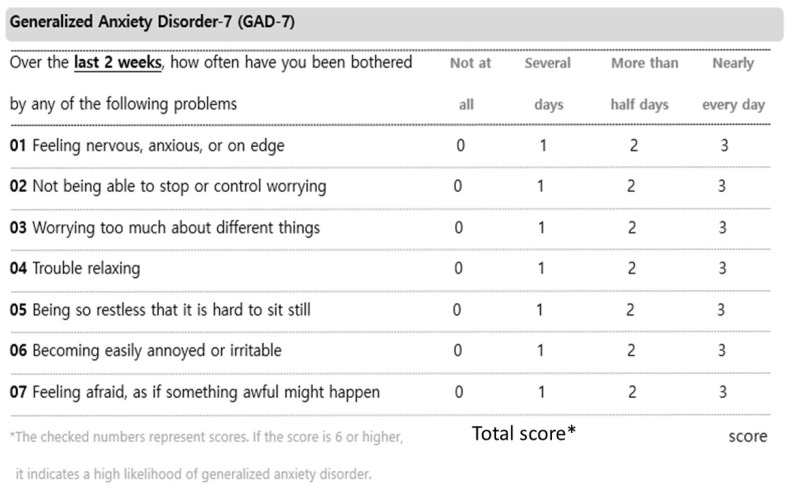
Generalized Anxiety Disorder-7.

This study encompasses five outcomes: impact, depression, anxiety, disability due to headache, and peak headache intensity. These outcomes were assessed using the Headache Impact Test-6 (HIT-6), Patient Health Questionnaire-9 (PHQ-9), Generalized Anxiety Disorder-7 (GAD-7), Migraine Disability Assessment (MIDAS), and a numeric rating scale (NRS) in peak, respectively. The items of each test, except for NRS, are detailed in Figure A1, Figure A2, Figure A3, Figure A4 and Figure A5.

The original project involved collecting video, speech, and ECG data from patients with headaches for the purpose of quantifying headache symptoms. After assessing five outcomes, video, speech, and ECG data were collected from all patients. However, although these data were collected, they have not undergone numerical processing. Therefore, the data cannot be used in this study.

In our study, we opted not to specify a predetermined sample size, as we believe that including as many participants as possible enhances the generalizability and statistical power of our machine learning findings, ensuring robust results. Therefore, we obtained approval from the Institutional Review Board for this issue because it was essential to ensure ethical conduct and participant safety. By maximizing participant inclusion, we aim to strengthen the validity and reliability of our study outcomes.

### 2.3. Data Source and Factors Associated with Headache

In the previous study, data were collected through surveys. The factors associated with headaches are investigated in Table 1.

**Table 1:** Survey Questions and Responses from Headache Patients.

Question	Answer
When did your headache start?	□ days ago, □ months ago, □ years ago
How long does the headache last? If you don’t take painkillers.	Within 30 min; More than 30 min to 4 h; More than 4 h~3 days; Three days or more
How often have you had headaches in the past 3 months?	1–11 days/year; 1–3 days/month; 1–2 days/week; 3–4 days/week or more than 15 days/month; newly occurred recently
How many days have you taken painkillers in the past month?	□ days [type: ]
What if you indicate the progress of your headache? Figure 1 illustrates the options for selecting headache course in the figure.	Figure 1.
Please indicate the intensity of your headache on a scale from 0 to 10.	0: No pain, 10: The most severe pain imaginable.
Do you experience any symptoms that are not visible during a headache or prior to its onset?	□ Yes □ No
Do you anticipate or foresee the occurrence of a headache before its onset?	If yes: □ hours before headache; Symptoms that can predict or anticipate the onset of a headache before it occurs: yawning, digestive disturbances, impaired concentration, mood changes, stiffness/pain in the neck, feeling of impending pain, fatigue, decreased appetite, sensitivity to sound, sensitivity to light, and others.
Please specify the symptoms youexperience while experiencing a headache.	Headache worsens if digestion is not smooth or if one feels nauseous. Increased sensitivity to noise during a headache. Headache intensifies with movement. Digestion becomes unsettled or one feels queasy during a headache. Increased sensitivity to smell during a headache. Dizziness during a headache. Vomiting during a headache. Increased sensitivity to light during a headache. Eyes appear congested with tears during a headache.
Which side of your head primarily hurts?	Right; left; both; alternatingly
Please indicate all factors that trigger your headaches.	Stress, fatigue, overeating, weekends, oversleeping, lack of sleep, noise, menstruation, colds, sexual activity, exercise, cold weather, hot weather, sunlight, change of seasons, upset stomach, hunger, alcohol, food, and others
Does anyone in your family experience headaches?	if yes: father, mother, children, sibling
Are you currently being treated for any other medical conditions?	□ No □ Yes If yes: The type of medical treatment received
Have you ever received psychiatric treatment for depression, anxiety, insomnia, or any other mental health condition?	□ No □ Yes If yes: The type of psychiatric treatment received

Based on the survey data and demographic information, we generated 86 features, including sex, age, height, weight, days since onset, headache duration, occurrence of new headaches, headache frequency per month, number of analgesics taken per month and per day, specific analgesics (such as acetaminophen, non-steroidal anti-inflammatory drugs, opioids, muscle relaxants, triptans, and antianxiety agents), pain patterns (1–6), preceding symptoms (such as yawning, digestive disorders, poor concentration, mood changes, stiff neck pain, sense of impending pain, fatigue, hypersensitivity to sound and light, heavy and uncomfortable head sensation, head tingling, dizziness, and nausea), headache symptoms (including headache triggered by indigestion, sensitivity to sound, worsened by movement, indigestion and nausea, sensitivity to smell, dizziness, vomiting, sensitivity to light, and tearfulness and eye congestion), headache location (right, left, both, middle, alternating), pulsation, trigger factors (such as stress, fatigue, overeating, weekend, oversleeping, lack of sleep, smells, sounds, menstruation, flu, sexual activity, exercise, cold, heat, sunlight, change in seasons, upset stomach, fasting, alcohol, and food), family history (father, mother, children, siblings), comorbidities (such as hypertension, diabetes, cardiovascular disease, stroke, dyslipidemia, cancer, depression, insomnia, anxiety, and panic disorder), and usual headache severity.

### 2.4. Data Preprocessing, Machine Learning and Statistics

#### 2.4.1. Data Preprocessing

Patients with missing values, or duplicate enrollments resulting from revisits, were excluded from the dataset. All data were coded numerically. Non-continuous data were categorized, while binary data were encoded using One-Hot encoding. Following data processing, the dataset was randomly split into training and testing sets with a ratio of 80:20 using the scikit learn package [30] in Python.

#### 2.4.2. Machine Learning and Statistics

We utilized four distinct machine learning algorithms along with conventional statistical analysis to predict HIT-6, MIDAS, PHQ-9, GAD-7, and peak NRS scores. These machine learning algorithms included Random Forest Regressor [31], Gradient Boosting Regressor [32], K-Neighbors Regressor [33], and Support Vector Regression [34], while Linear Regression served as the conventional statistical analysis method.

Random Forest Regressor is one of the powerful regression algorithms used in machine learning. It is fundamentally constructed using the bagging (Bootstrap Aggregating) method [35]. Multiple decision trees are trained independently, and their individual predictions are combined to make more accurate predictions. During the construction of each tree, randomly selected features are used for splitting, which increases model diversity and helps prevent overfitting. Random Forest is particularly useful when datasets have many or diverse features, and it generally exhibits stable and robust performance compared to other regression algorithms.

Gradient Boosting Regressor is one of the ensemble learning methods. This algorithm performs predictions using multiple decision trees and sequentially trains new trees to complement the errors of previous trees [36,37]. As each tree operates to mitigate the errors of the preceding tree, Gradient Boosting Regressor typically exhibits high performance. Moreover, it can control the complexity of the model or trees to reduce overfitting by adjusting parameters. This algorithm is applicable to various types of datasets, particularly useful when there are interactions among features or nonlinear relationships.

The K-Neighbors Regressor is a regression algorithm that predicts based on the values of the nearest neighbors in the given dataset. This algorithm identifies the K-nearest neighbors around each data point in the dataset and averages their values to use as the prediction for that data point [38]. It performs effectively, particularly when the data are dense and exhibit regular patterns.

Support Vector Regression (SVR) is one of the machine learning algorithms used to solve regression problems. SVR learns the patterns of given data and is used to predict continuous values based on them [39]. SVR is a variation of Support Vector Machine (SVM) and works by mapping data points into a high-dimensional space to find linear or nonlinear decision boundaries. It utilizes the concept of support vectors to find the decision boundaries. SVR optimizes by selecting as many data points as possible as support vectors within a certain margin.

After training the prediction models using the training set, we evaluated their performance on the test set using evaluation metrics such as root mean squared error (RMSE), mean squared error (MSE), mean absolute error (MAE), and R-squared (R^2^).

The R^2^ coefficient of determination is a metric in regression analysis that indicates how well the model fits the given data, representing the proportion of the total variance in the dependent variable explained by the model. A value of R^2^ close to 1 indicates that the model adequately explains the data, while a value close to 0 suggests that the model fails to explain the data. Negative values indicate that the model poorly describes the data [40].

MAE represents the average of the absolute errors between the actual and predicted values [41]. It measures the discrepancy between the predicted and actual values by taking the absolute difference for each data point and computing the mean of these differences. In simple terms, a smaller MAE implies more accurate predictions by the model. Since MAE indicates how much, on average, predictions deviate from the actual values, lower values are interpreted as the model performing better predictions.

MSE calculates the squared error between the predicted and actual values for each data point and then computes the average of these squared errors [42]. In simple terms, MSE is the average of the squared differences between predicted and actual values. A smaller MSE indicates more accurate predictions by the model. Since MSE squares the prediction errors, it gives higher weights to larger errors while ignoring smaller ones.

RMSE represents the square root of MSE [43]. In other words, it calculates the squared errors between predicted and actual values for each data point, computes the average of these squared errors, and then takes the square root of this value. Essentially, RMSE is the square root of MSE, serving as a metric to express the model’s prediction errors in the same units as the actual values. A smaller RMSE implies more accurate predictions by the model, as it reflects the square root of the average prediction error, allowing for an intuitive understanding of the magnitude of prediction errors.

Machine learning and model evaluation were conducted using Google Colab (Python version 3.10.12; Mountain View, CA, USA). For descriptive statistics, continuous features were summarized using medians and interquartile ranges, while categorical features were presented with counts and corresponding percentages. Disparities between groups were quantified using absolute standardized differences. Linear regression and absolute standardized differences were computed using R (version 4.2.3, R Foundation for Statistical Computing, www.r-project.org).

#### 2.4.3. Feature Importance

We utilized the Shapley value method to elucidate feature importance in machine learning models. Derived from game theory, the Shapley value is employed to assess contributions, akin to understanding the role of each player in a game [44]. In the realm of machine learning, this technique is leveraged to delineate the extent to which each feature impacts the model’s predictions, facilitating insights into pivotal features for prediction elucidation. The Shapley value provides an accurate estimation of each feature’s contribution, factoring in interactions among features [45]. In traditional linear regression analysis, we examined feature importance through estimated coefficients and *p*-values. We identified features based on a threshold *p*-value of less than 0.05.

## 3. Results

### 3.1. Patient Characteristics

**Figure 2:**
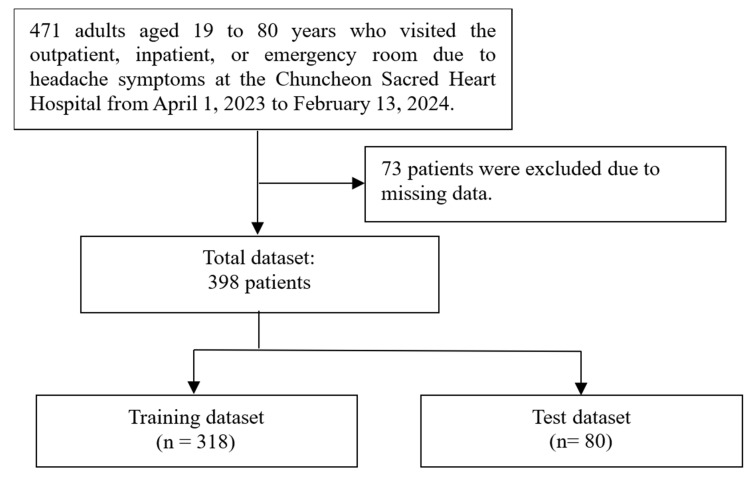
This flowchart illustrates the process of dividing the dataset for the study.

The initial dataset for this study consisted of 471 cases documented between 1 April 2023 and 13 February 2024. In total, 73 cases were excluded due to missing data, and a total of 398 cases were available for analysis. These cases were subsequently split into a training set (*n* = 318) and a test set (*n* = 80). The process is illustrated in Figure 2. Absolute standardized differences between the training set and test set exceeded 0.25 for head tingling, dizziness (as preceding symptoms), cold, and food (as trigger factors). Conversely, for other features, the ASD between the training set and test set remained below 0.25. The demographic and headache-related characteristics of both the training and test sets are summarized in Table 2.

#### Analgesics

**Figure 3:**
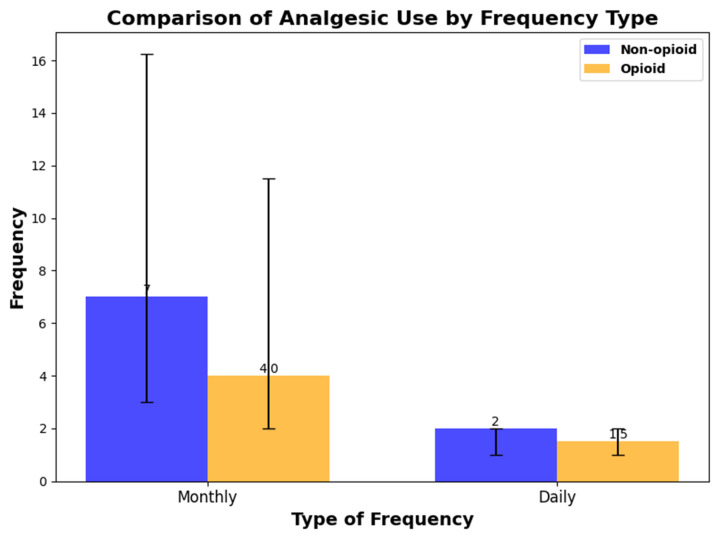
The monthly frequency and daily dose frequency of analgesic use among participants taking only non-opioid analgesics and those taking opioid analgesics.

In a cross-sectional analysis of analgesic use, 310 out of 398 participants were taking analgesics. The median frequency of analgesic use over a month was 4 days (IQR: 2 to 12), and the median number of daily doses was 1.5 (IQR: 1 to 2). A total of 186 participants used acetaminophen, while 171 used non-steroidal anti-inflammatory drugs, and 72 participants used both. Additionally, 30 participants were taking opioid analgesics. The monthly frequency and daily dose frequency of analgesic use among participants taking only non-opioid analgesics and those taking opioid analgesics are summarized in Figure 3.

### 3.2. Prediction of HIT-6, MIDAS, PHQ-9, GAD-7, and Peak NRS

The initial assessment encompassed 86 features detailed in Table 1, excluding HIT-6, MIDAS, PHQ-9, GAD-7, and peak NRS. Utilizing these 86 features, a model was constructed to predict each target and evaluated using the training set. In the evaluation of HIT-6, the Random Forest model demonstrated superior performance across all evaluation metrics. For MIDAS, the Support Vector Regressor model outperformed others, achieving the best performance in terms of RMSE, MAE, and MAE. Similarly, in the assessment of PHQ-9, the Random Forest model exhibited the best performance across all evaluation metrics. In the case of GAD-7, the Random Forest model showcased the best performance in terms of RMSE, MSE, and R^2^. Lastly, for peak NRS, the Random Forest model consistently demonstrated the best performance across all evaluation metrics (Table 3).

### 3.3. Important Features

#### 3.3.1. SHAP Values

**Figure 4:**
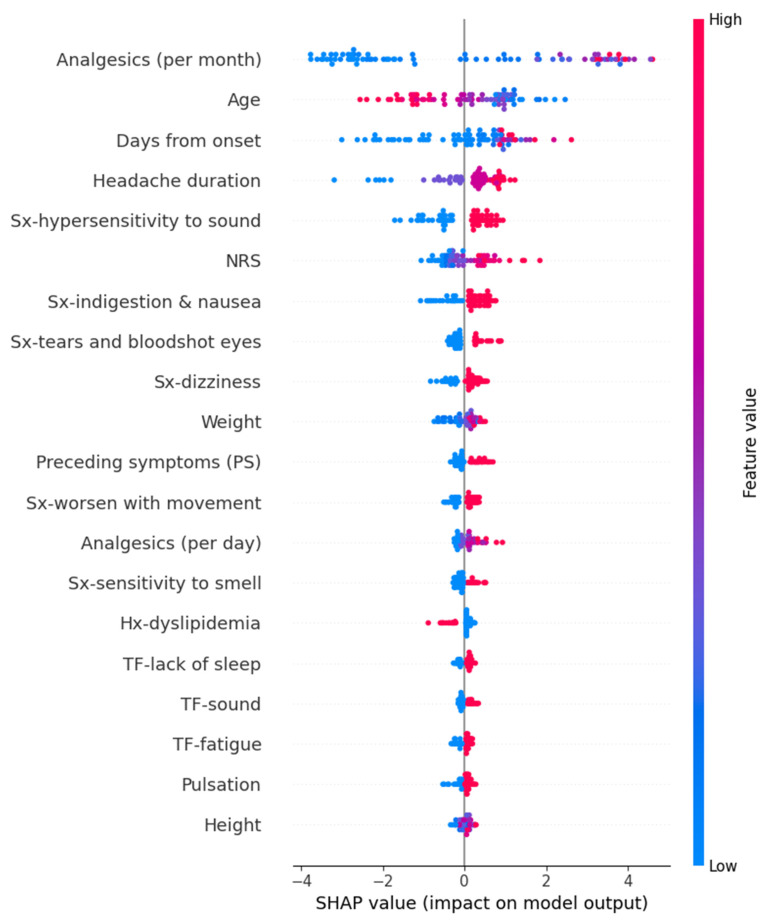
SHAP value in Random Forest model of predicting Headache Impact Test-6. Hx, history; NRS, numeric rating scale as usual headache intensity; SHAP, Shapley; Sx, symptom; TF, trigger factor.

We calculated SHAP values for the models that exhibited the best performance for each target variable. In the Random Forest model for predicting HIT-6 scores, variables such as high frequency per month of analgesics, young age, long duration from onset, headache duration, hypersensitivity to sound, high intensity of usual headache, and others contributed positively to the prediction values. The detailed SHAP values of these features are summarized in Figure 4.

**Figure 5:**
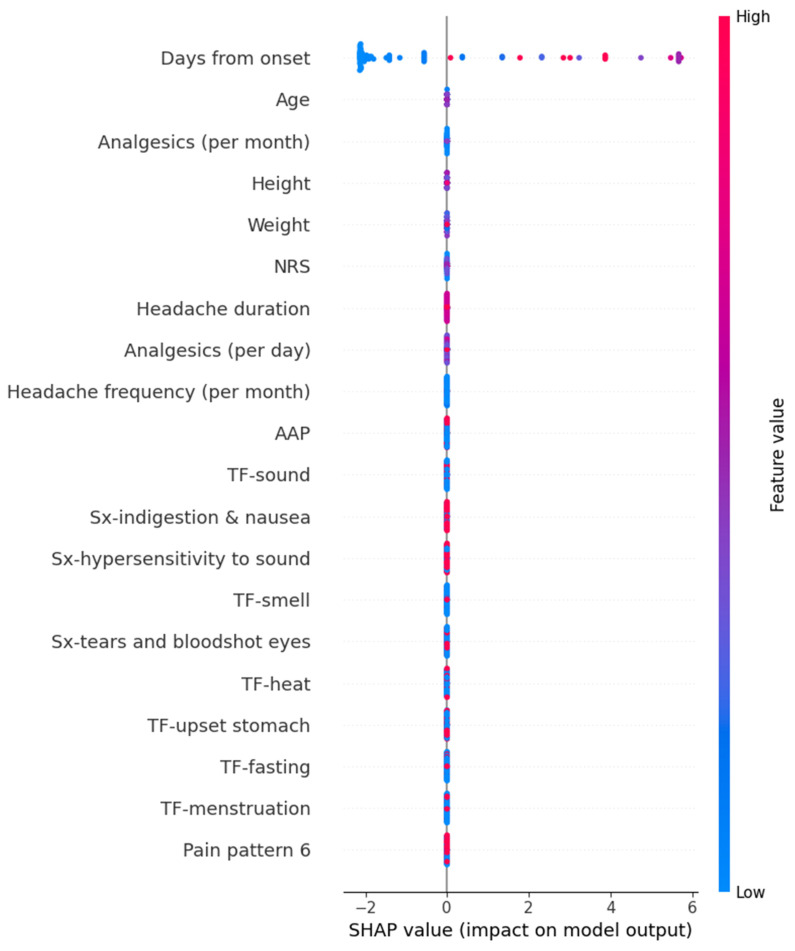
SHAP value in Supportive vector regressor model predicting Migraine Disability Assessment. AAP, acetaminophen; NRS, numeric rating scale as usual headache intensity; SHAP, Shapley; Sx, symptom; TF, trigger factor.

In the SVR model for predicting MIDAS scores, a short duration from onset exhibited a decreasing effect on the prediction values. However, the other features did not show a significant directional trend in SHAP values. The detailed SHAP values of these features are summarized in Figure 5.

**Figure 6:**
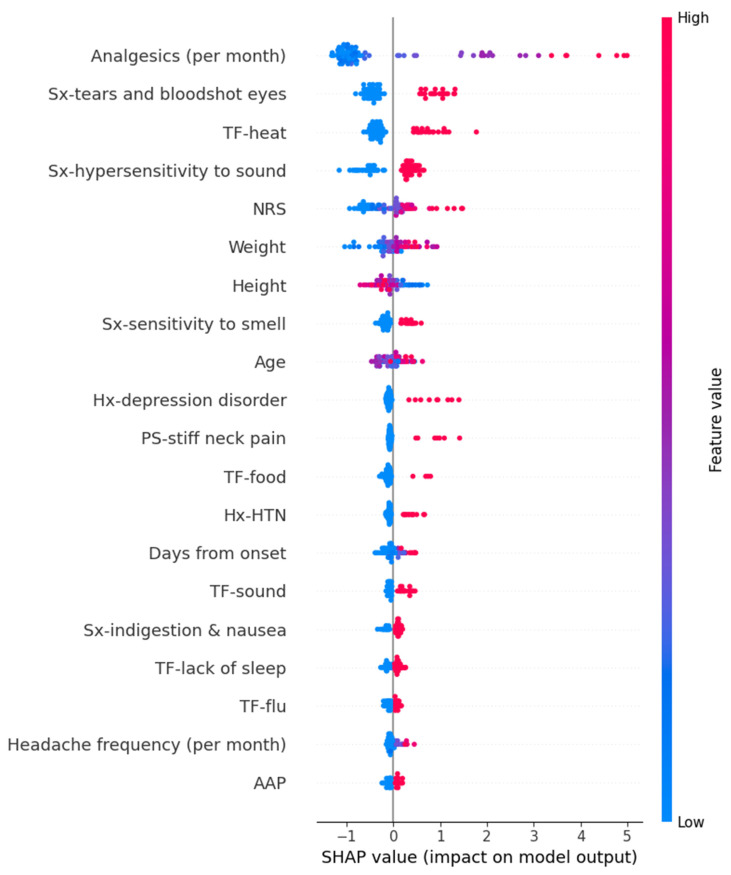
SHAP value in Random Forest model of predicting Patient Health Questionnaire-9. AAP, acetaminophen; HTN, hypertension; Hx, history; NRS, numeric rating scale as usual headache intensity; PS, preceding symptom; SHAP, Shapley; Sx, symptom; TF, trigger factor.

In the Random Forest model for predicting PHQ-9 scores, variables such as high frequency per month of analgesics, tears and bloodshot eyes, heat as a trigger factor, hypersensitivity to sound, high intensity of usual headache, and others contributed increasing the prediction values. The detailed SHAP values of these features are summarized in Figure 6.

**Figure 7:**
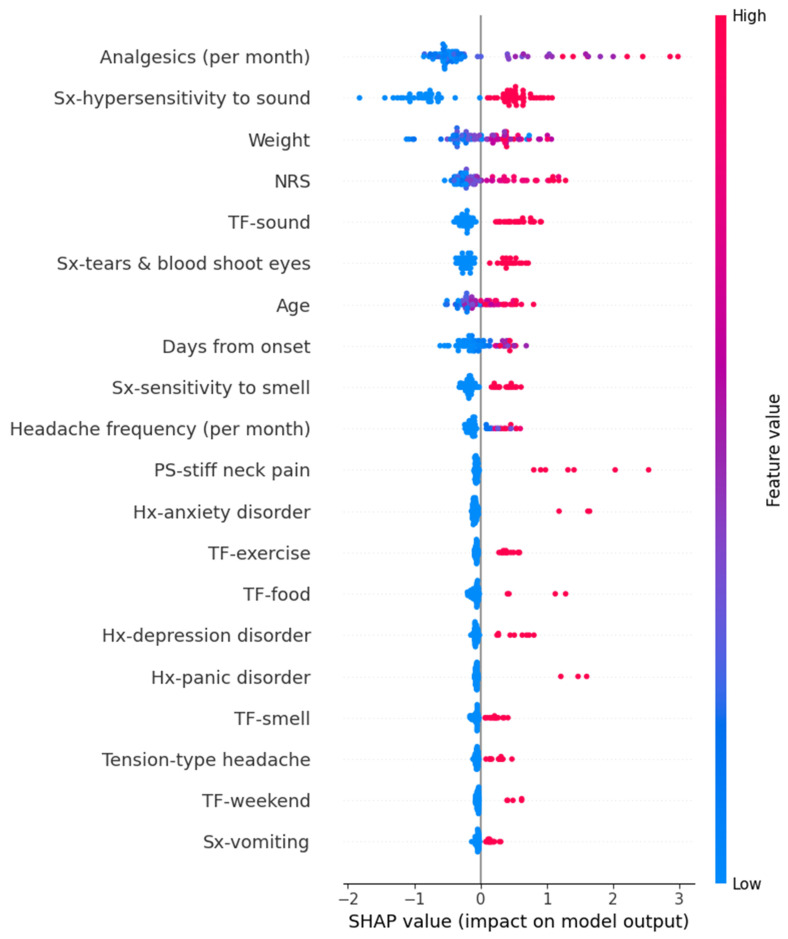
SHAP value in Random Forest model of predicting Generalized Anxiety Disorder-7. Hx, history; NRS, numeric rating scale as usual headache intensity; PS, preceding symptom; SHAP, Shapley; Sx, symptom; TF, trigger factor.

In the Random Forest model for predicting GAD-7 scores, variables such as high frequency per month of analgesics, hypersensitivity to sound, heavy weight, high intensity of usual headache, sound as a trigger factor, and others contributed to an increase in the prediction values. The detailed SHAP values of these features are summarized in Figure 7.

**Figure 8:**
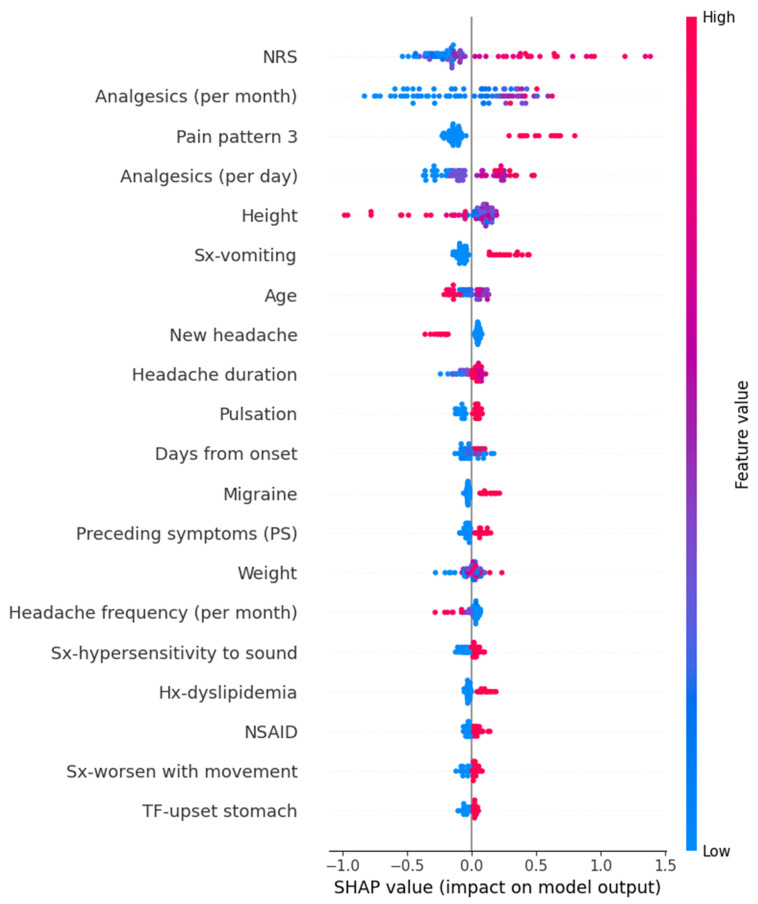
SHAP value in Random Forest model of predicting peak headache intensity. Hx, history; NRS, numeric rating scale as usual headache intensity; NSAID, non-steroidal anti-inflammatory drug; SHAP, Shapley; Sx, symptom; TF, trigger factor.

In the Random Forest model for predicting peak headache intensity, variables such as high frequency per month of analgesics, high intensity of usual headache, a course of increasing intensity, high frequency per day of analgesics, tall height, and others contributed to an increase in the prediction values. The detailed SHAP values of these features are summarized in Figure 8.

#### 3.3.2. Coefficients and *p* Value in Linear Regression Models

The linear regression models for HIT-6, MIDAS, PHQ-9, GAD-7, and peak NRS had 8, 16, 13, 7, and 4 features with *p*-values < 0.05, respectively. The estimated coefficients and *p*-values of each feature in each model are summarized in Table 4. The overlapping features between linear regression and machine learning models were five, four, eight, five, and four for HIT-6, MIDAS, PHQ-9, GAD-7, and peak NRS, respectively. All overlapping features in HIT-6, PHQ-9, GAD-7, and peak NRS exhibited the same direction of influence on the prediction values in both linear regression models and machine learning models. However, SHAP values of the SVR model for MIDAS showed a significant pattern only for one feature.

## 4. Discussion

This study investigated the psychological impact and functional impairment of headache-associated factors using both machine learning and conventional statistics. A total of 398 cases were analyzed, with 318 cases assigned to the training set and 80 cases to the test set. Utilizing 86 features excluding HIT-6, MIDAS, PHQ-9, GAD-7, and peak NRS, models were constructed and evaluated. The Random Forest model demonstrated superior performance for predicting HIT-6, PHQ-9, GAD-7, and peak headache intensity, while the Support Vector Regressor outperformed for MIDAS prediction. SHAP values were utilized to identify influential features for each target variable. Overlapping features between linear regression and machine learning models were observed for HIT-6, PHQ-9, GAD-7, and peak NRS, indicating a consistent directional influence on prediction values across both types of models. However, no overlapping features were found for MIDAS prediction.

The correlation between headaches and mental health issues is widely acknowledged, with depression, anxiety, and physical ailments often accompanying such conditions [46,47]. Previous research has extensively examined the factors contributing to mental health effects and the onset of functional disorders using conventional statistical analyses [48]. Specifically, in chronic headache cases, symptoms associated with anxiety and depression tend to be more prevalent among patients with chronic migraines. The frequency of headache episodes, headache history, and gender are correlated with both anxiety and depression. Additionally, the risk of these mental health conditions increases in patients with a history of drug overdose and with preceding symptoms of migraines [49,50]. In patients with headaches, abusing pain relievers diminishes their quality of life in aspects related to health, such as physical function and pain perception [51]. However, there is no evidence suggesting that an increase in the duration of headaches leads to an increase in anxiety. As mentioned earlier, prior research has demonstrated the close association between headache-related factors and quality of life, depression, and anxiety. These factors have also emerged as significant features in our machine learning models, indicating their importance in patient-specific evaluations employing machine learning techniques.

Recently, several studies have been reported that apply machine learning to headache patients. Kwon J and colleagues reported headache classification using machine learning and patient-reported questionnaires [52]. Stubberud A and colleagues predicted migraine using data from mobile phone diaries and wearables [53]. Patrizia Ferroni P and colleagues predicted medication abuse in migraine patients [54]. Yang F and colleagues classified primary and secondary headaches using complete blood count and machine learning [55]. However, these studies differ from ours as they focused on developing classification models. In contrast, our study developed a regression model. Additionally, we identified the effect of features included in each model on outcome variables such as quality of life, anxiety, depression, and maximum pain intensity through the regression model. By focusing on the relationship between features and outcomes rather than solely on outcome prediction, our approach highlights the importance of understanding these relationships in a personalized approach. This approach can help optimize treatment and management methods by considering individual characteristics and circumstances of the patient. Examining the relationship between outcome and features using a regression model of machine learning can provide a more accurate understanding of the effects of each feature on the outcome and aid in developing an optimal treatment plan tailored to individual characteristics.

While some common features were identified between the machine learning and linear regression models, several crucial features highlighted by the high-performing machine learning models did not appear as significant in the linear regression model. For instance, although the duration since onset is a vital feature in the high-class models of HIT-6 and MIDAS, reflecting headache-related disability, it did not yield significant coefficients in the linear regression models for HIT-6 and MIDAS. However, previous studies have indicated that chronic headaches are associated with higher MIDAS and HIT-6 scores [56,57]. Similarly, the symptom of hypersensitivity to sound emerged as a significant feature in the high-class models of PHQ-9 and GAD-7, representing depression and anxiety, respectively, but was absent in the linear regression model. To our knowledge, no study has reported the effect of hypersensitivity to sound on depression and anxiety. These features, unique to machine learning models, and the differing features between machine learning and conventional statistical models offer a novel perspective on determining essential headache-related factors that influence the degree of headache-related disability, depression, and anxiety.

In our study, the frequency of analgesic medication per month emerged as the most impactful feature in the best models of HIT-6, PHQ-9, GAD-7, and peak headache intensity. Additionally, linear regression models of HIT-6, MIDAS, PHQ-9, and GAD-7 demonstrated significant coefficients with *p* < 0.05. These results suggest that frequent analgesic medication is associated with poorer life quality, increased depression, and anxiety. This finding aligns with previous studies indicating that drug overdose can exacerbate mental health conditions [49,50,51]. However, our models did not distinguish whether the medication itself or the severity of pain prompting the medication use contributed to these outcomes. Given the substantial influence of general headache intensity on SHAP values across all target outcomes, further investigation is warranted to delineate the specific contributions of medication usage and pain severity to these associations.

When attempting to derive feature importance using SHAP in the SVR model, the absence of a clear pattern may be attributed to the inherent complexity of SVR. SVR employs nonlinear functions, determined by kernel functions and hyperparameters, to transform and model the data [58]. This complexity can obscure the understanding of how the SVR model generates prediction results, as SVR considers intricate interactions among various features, making it challenging to pinpoint the most significant ones. Consequently, SHAP values may exhibit irregular scattering or lack discernible patterns. Moreover, unlike linear regression, which utilizes coefficients to directly gauge the influence of each feature, SVR presents additional complexities in interpreting feature importance through SHAP.

The present study employs a comprehensive approach, utilizing both traditional statistical methods and machine learning techniques to explore the relationship between headache-related factors and psychological impact, as well as functional impairment. This multifaceted approach enables a deeper understanding of the intricate interactions among variables. However, several limitations should be noted. Firstly, due to its retrospective nature, the study is unable to establish causality between headache-related factors and psychological impact, highlighting the need for future prospective studies. Secondly, the lack of external validation for the prediction models undermines the assessment of their generalizability and robustness across different populations or settings. Third, the study included the subjective nature of data obtained through patient recall or memory-based surveys. This may lead to inaccuracies or distortions in the information, potentially resulting in distorted predictions by the model. Lastly, there is a possibility that not all potential confounding variables influencing the relationship between headache-related factors and psychological impact were accounted for, potentially introducing bias into the results. In particular, antidepressants such as selective serotonin reuptake inhibitors can induce headaches [59]; however, our data do not include detailed information about antidepressants, which may act as a confounding variable.

## 5. Conclusions

In conclusion, our study utilized both traditional statistical methods and machine learning techniques to investigate the relationship between headache-related factors and psychological impact, as well as functional impairment. Our findings highlight the importance of considering individual characteristics and circumstances in the evaluation and management of headache patients. While some common features were identified between machine learning and linear regression models, several crucial features highlighted by machine learning models did not appear as significant in linear regression. These differences offer a new perspective on determining essential headache-related factors that influence the degree of disability, depression, and anxiety. To enhance the generalizability of our results, an increase in the sample size and expansion of the sampling regions are required.

## Figures and Tables

**Figure 1:**
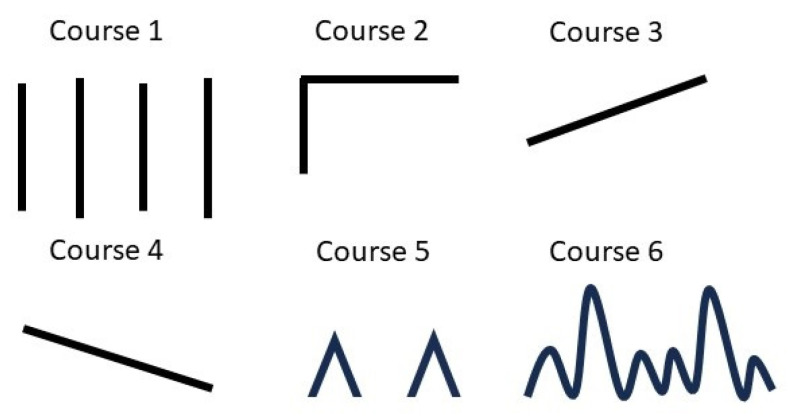
Options for selecting headache course.

**Table 2:** Demographic and headache-associated features according to training set (*n* = 318) and test set (*n* = 80).

Features	Train	Test	ASD	Features	Train	Test	ASD	Features	Train	Test	ASD
Sex (Female)	209 (66)	58 (73)	0.15	PS-fatigue	10 (3)	3 (4)	0.03	TF-sex	10 (3)	4 (5)	0.09
Age	49 (37.25, 60)	46 (34, 62)	0.04	PS-hypersensitivity to sound	5 (2)	0 (0)	0.14	TF-exercise	54 (17)	16 (20)	0.08
Height	162 (158, 170)	163 (157.75, 169)	0.01	PS-sensitive to light	5 (2)	1 (1)	0.03	TF-cold	101 (32)	15 (19)	0.33
Weight	63 (54, 73)	62.5 (54, 72.25)	0	PS-heavy and stuffy and uncomfortable head	36 (11)	11 (14)	0.07	TF-heat	99 (31)	23 (29)	0.05
Days from onset	180 (14, 2190)	255 (15.5, 1825)	0.04	PS-head tingling	13 (4)	1 (1)	0.26	TF-sunlight	94 (30)	22 (28)	0.05
Migraine	59 (19)	19 (24)	0.12	PS-dizziness	13 (4)	1 (1)	0.26	TF-change in seasons	83 (26)	21 (26)	0
Tension-type headache	63 (20)	15 (19)	0.02	PS-feel nausea	7 (2)	1 (1)	0.09	TF-upset stomach	167 (53)	42 (53)	0
Headache duration	3 (2, 4)	3 (2, 3.25)	0.01	Sx-headache when indigestion	174 (55)	45 (56)	0.03	TF-fasting	42 (13)	18 (23)	0.22
New headache	60 (19)	12 (15)	0.11	Sx-hypersensitivity to sound	197 (62)	48 (60)	0.04	TF-alcohol	121 (38)	25 (31)	0.15
Headache frequency per month	1 (1, 2)	1 (1, 1.5)	0.05	Sx-worsen with movement	197 (62)	52 (65)	0.06	TF-food	47 (15)	4 (5)	0.45
Analgesics per month	3 (1, 10)	4 (1, 10)	0.02	Sx-indigestion & nausea	210 (66)	58 (73)	0.14	Family Hx-father	16 (5)	3 (4)	0.22
Analgesics per day	1 (1, 2)	1 (1, 2)	0.01	Sx-sensitivity to smell	101 (32)	18 (23)	0.22	Family-Hx mother	69 (22)	17 (21)	0.08
Acetaminophen	151 (47)	35 (44)	0.08	Sx- dizziness	223 (70)	57 (71)	0.02	Family Hx-children	21 (7)	6 (8)	0.07
NSAID	137 (43)	34 (43)	0.01	Sx-vomiting	63 (20)	21 (26)	0.15	Family Hx-sibling	22 (7)	6 (8)	0.08
Opioid	24 (8)	6 (8)	0	Sx-hypersensitivity to light	138 (43)	31 (39)	0.1	Hx-HTN	66 (21)	14 (18)	0.09
Muscle relaxant	53 (17)	11 (14)	0.08	Sx- tears and bloodshot eyes	98 (31)	24 (30)	0.02	Hx-DM	30 (9)	9 (11)	0.06
Triptan	25 (8)	8 (10)	0.07	Headache site right	88 (28)	19 (24)	0.09	Hx-cardiac disease	13 (4)	5 (6)	0.09
Antianxiety	26 (8)	3 (4)	0.23	Headache site left	93 (29)	27 (34)	0.1	Hx-CVA/TIA	16 (5)	2 (3)	0.16
Antidepressants	7 (2)	5 (6)	0.16	Headache site both	106 (33)	21 (26)	0.16	Hx-dyslipidemia	55 (17)	16 (20)	0.07
Headache course 1	28 (9)	4 (5)	0.17	Headache site middle part	20 (6)	3 (4)	0.13	Hx-cancer	11 (3)	1 (1)	0.2
Headache course 2	76 (24)	22 (28)	0.08	Headache site taking turns	25 (8)	10 (13)	0.14	Hx-depression disorder	39 (12)	9 (11)	0.03
Headache course 3	68 (21)	13 (16)	0.14	Pulsation	200 (63)	55 (69)	0	Hx-insomnia	22 (7)	9 (11)	0.14
Headache course 4	15 (5)	5 (6)	0.06	TF-stress	253 (80)	64 (80)	0.01	Hx-anxiety disorder	14 (4)	3 (4)	0.03
Headache course 5	32 (10)	6 (8)	0.1	TF-fatigue	218 (69)	56 (70)	0.03	Hx-panic disorder	11 (3)	3 (4)	0.02
Headache course 6	149 (47)	44 (55)	0.16	TF-overeating	49 (15)	12 (15)	0.01	NRS	2 (0, 4)	2 (1, 5)	0.01
Preceding symptoms	120 (38)	27 (34)	0.08	TF-weekend	24 (8)	5 (6)	0.05	HIT-6	57 (50, 63)	58 (50, 63)	0.03
PS-yawn	3 (1)	0 (0)	0.11	TF-oversleep	47 (15)	11 (14)	0.03	MIDAS	6.5 (1, 20)	8 (2, 20)	0.08
PS-Digestive disorders	6 (2)	0 (0)	0.16	TF-lack of sleep	179 (56)	50 (63)	0.13	PHQ-9	7.5 (4, 12)	8 (5, 11)	0.01
PS-difficulty concentrating	1 (0)	0 (0)	0.06	TF-smell	64 (20)	15 (19)	0.04	GAD-7	4.5 (2, 9)	4 (2, 8)	0.09
PS-mood changes	3 (1)	4 (5)	0.19	TF- sound	103 (32)	25 (31)	0.02	NRS peak	7 (5, 8)	7 (5,8)	0.23
PS-stiff neck/pain	23 (7)	7 (9)	0.05	TF-menstruation	46 (14)	15 (19)	0.11				
PS-a sense of impending pain	10 (3)	2 (3)	0.04	TF-flu	161 (51)	33 (41)	0.19				

Continuous data were expressed as a median and interquartile range, and categorical data were expressed as a number and percentage. ASD, absolute standardized difference; CVA, cerebral vascular accident; DM, diabetes mellitus; GAD, Generalized Anxiety Disorder; HIT, Headache Impact Test; Hx, history; HTN, hypertension; MIDAS, Migraine Disability Assessment; NSAID, non-steroidal anti-inflammatory drug; NRS, numeric rating scale; PHQ, Patient Health Questionnaire; PS, preceding symptom; Sx, symptom; TF, trigger factor; TIA, transient ischemic attack.

**Table 3:** The performance metrics, including RMSE, MSE, MAE, and R^2^ for HIT-6, MIDAS, PHQ-9, GAD-7, and peak NRS in patients with headaches. GAD, Generalized Anxiety Disorder; HIT, Headache Impact Test; MIDAS, Migraine Disability Assessment; MAE, mean absolute error; Migraine Disability Assessment; MSE, mean squared error; NRS, numeric rating scale; PHQ, Patient Health Questionnaire; RMSE, root mean squared error; ^†^: the lowest value in RMSE, MSE, and MAE; ^‡^: the positive highest value in R^2^.

1	Algorithm	RMSE	MSE	MAE	R^2^
HIT-6	Random Forest	7.624 ^†^	58.119 ^†^	6.011 ^†^	0.157 ^‡^
	Gradient boosting	8.014	64.225	6.402	0.068
	K-Neighbors regressor	8.383	70.275	6.713	−0.019
	Supportive vector regressor	7.967	63.479	6.629	0.079
	Linear regression	8.607	74.082	6.729	−0.074
MIDAS	Random Forest	22.951	526.738	16.33	−0.241
	Gradient boosting	29.884	893.035	19.054	−1.104
	K-Neighbors regressor	23.08	532.699	16.27	−0.255
	Supportive vector regressor	22.553 ^†^	508.64 ^†^	13.86 ^†^	−0.199 ^‡^
	Linear regression	23.72	562.662	18.07	−0.326
PHQ-9	Random Forest	5.026 ^†^	25.263 ^†^	3.641 ^†^	0.144 ^‡^
	Gradient boosting	5.599	31.352	4.18	−0.062
	K-Neighbors regressor	5.955	35.467	4.553	−0.201
	Supportive vector regressor	5.536	30.648	4.204	−0.038
	Linear regression	5.913	34.963	4.582	−0.184
GAD-7	Random Forest	4.886 ^†^	23.872 ^†^	3.706	0.053 ^‡^
	Gradient boosting	5.086	25.87	3.83	−0.027
	K-Neighbors regressor	5.267	27.736	3.878	−0.101
	Supportive vector regressor	5.04	25.399	3.642 ^†^	−0.008
	Linear regression	5.393	29.086	4.143	−0.154
Peak NRS	Random Forest	1.723 ^†^	2.968 ^†^	1.417 ^†^	0.107 ^‡^
	Gradient boosting	1.82	3.311	1.459	0.003
	K-Neighbors regressor	1.927	3.714	1.607	−0.118
	Supportive vector regressor	1.816	3.296	1.498	0.008
	Linear regression	2.162	4.673	1.759	−0.406

**Table 4:** The estimated coefficients and *p*-values of each feature in linear regression models of HIT-6, MIDAS, PHQ-9, GAD-7, and peak NRS. The features highlighted in bold are those commonly selected through Shapley values by the machine learning models exhibiting the best performance.

Target	Feature	Coefficient	*p* Value	Target	Feature	Coefficient	*p* Value
HIT-6	**Age**	−0.09	0.01	PHQ-9	**Height**	−0.11	0.042
	**Headache duration**	1.03	0.005		Tension-type headache	1.77	0.017
	New headache	−2.97	0.004		Headache duration	0.79	0.003
	**Analgesics (per month)**	0.25	<0.001		**Analgesics (per month)**	0.12	0.002
	**Sx-tears and blood shoot eyes**	2.24	0.011		Preceding symptoms (PS)	−2.09	0.04
	TF-flu	−2.04	0.014		PS-Digestive disorders	5.55	0.022
	Hx-panic disorder	−5.01	0.016		**PS-stiff neck pain**	3.89	0.002
	**NRS**	0.64	<0.001		PS-dizziness	3. 44	0.034
MIDAS	Sex	−13.74	0.002		**Sx-sensitivity to smell**	1.47	0.032
	**Age**	−0.32	0.009		**Sx-tears and blood shoot eyes**	1.75	0.006
	New headache	−7.43	0.03		**TF-sound**	1.62	0.02
	**Analgesics (per month)**	0.88	<0.001		**TF-heat**	1.92	0.005
	PS-stiff neck pain	13.59	0.021		**NRS**	0.28	0.027
	PS-head tingling	18.27	0.013	GAD-7	**Weight**	0.05	0.038
	PS-dizziness	21.9	0.003		**Analgesics (per month)**	0.07	0.049
	**Sx-tears and bloodshot eyes**	9.42	0.001		Triptan	−2.26	0.046
	TF-menstruation	10.55	0.007		**PS-stiff neck pain**	2.95	0.019
	TF-sex	−18.74	0.006		**TF-exercise**	1.47	0.043
	TF-exercise	6.7	0.05		**TF-food**	1.88	0.031
	TF-cold	8.82	0.003		Family Hx-mother	1.6	0.014
	Hx-HTN	8	0.016	Peak NRS	**New headache**	−0.85	0.005
	Hx-depression disorder	9.38	0.025		**Pain pattern 3**	1.38	<0.001
	Hx-anxiety disorder	−13.84	0.027		**Hx-dyslipidemia**	0.71	0.025
	**NRS**	1.6	0.006		**NRS**	0.27	<0.001

GAD-7, Generalized Anxiety Disorder-7; HIT-6, Headache Impact Test-6; HTN, hypertension; Hx, history of comorbid disease; MIDAS, Migraine Disability Assessment; NRS, numeric rating scale as usual headache intensity; PHQ-9, Patient Health Questionnaire-9; Sx, symptom; TF, trigger point.

## Data Availability

The datasets presented in this article are not readily available because the study is ongoing, and data collection is still in progress. Requests to access the datasets should be directed to the corresponding author’s email.
